# Investigating Anxiety and Fear of COVID-19 as Predictors of Internet Addiction With the Mediating Role of Self-Compassion and Cognitive Emotion Regulation

**DOI:** 10.3389/fpsyt.2022.841870

**Published:** 2022-03-23

**Authors:** Reihaneh Moniri, Kimia Pahlevani Nezhad, Fahimeh Fathali Lavasani

**Affiliations:** Deputy of Behavioral Sciences and Mental Health, Tehran Psychiatric Institute, Iran University of Medical Sciences, Tehran, Iran

**Keywords:** anxiety, COVID-19, internet addiction, self-compassion, cognitive emotion regulation

## Abstract

**Background:**

In addition to many deaths due to the Coronavirus pandemic, many psychological issues and problems are affecting people's health. Including the constant anxiety and fear of infecting themselves and their families, COVID-19 has led to excessive spending of time in cyberspace and the Internet.

**Methods:**

In this study, the role of fear and anxiety of COVID-19 in predicting Internet addiction among 1,008 students was investigated. The mediating role of the two components of self-compassion and cognitive emotion regulation has also been measured. Data collection was done online due to the outbreak of the disease and a modeling method was used to analyze the data.

**Results:**

The results shows that anxiety and fear of COVID-19 has a positive and significant relationship with both Internet addiction (*r* = 0.32) and maladaptive cognitive emotion regulation strategies (*r* = 0.17), and it has a negative relationship with self-compassion (*r* = −0.25).

**Conclusions:**

The findings suggest that self-compassion can play a protective role against internet addiction at the time of COVID-19 pandemic while maladaptive strategies for emotion regulation can be risk factors for anxiety and fear of the virus.

## Introduction

In December 2019, a virus of unknown origin entangled the world called SARS-CoV-2, or more commonly referred to as the COVID-19 virus. The virus began to spread from China and the city of Wuhan, which spread widely around the world despite China's rapid quarantine efforts (1). According to the World Health Organization (WHO), the coronavirus pandemic has become a global concern and measures such as social distancing, regular hand washing, and in a case of infection, house quarantining for 7 to 14 days is necessary (2). Based on WHO, the number of confirmed cases worldwide is more than 373 million and the number of deaths is about 5.65 million people as of January 2022, and is on an ascending path. In particular, Iran has reported about 6.34 million confirmed cases and more than 132 thousand deaths (3). The pandemic has effected almost every part of human life (4) such as: socializing, working, planning and even shopping. Also the social isolation which is one of the consequences of the pandemic, has not only changed the lifestyles of the people all over the world, such as the quantity of physical activities and sleep patterns, it has also influenced mental health and emotional responses of the people (5). Even it is studied that less physical activity, sleep problems related to the quarantine, and internet usage can be the risk factors for increased anxiety at the time of pandemic (6). Some psychological impacts of the disease have been investigated with the onset of the prevalence but the solutions in order to reduce the damage have been somehow neglected (7). Fear and anxiety caused by little knowledge about the virus (8), fear of disease and death (2), spreading false news (9), reduced social contacts (1), restrictions on the use of public transportations (10), economic problems (11) and excessive use of social media (12) are among the problems of this period of time. Dr. David Murphy (president of the British Psychological Society) introduced fear and anxiety as one of the basic variables that should be investigated during COVID-19 pandemic (13). Besides that, fear and anxiety as consequences of COVID-19 can lead to disorders such as depression and anxiety among adolescents (14).

An unavoidable requirement of the coronavirus pandemic is observing physical and social distancing. Physical distancing means staying 6 feet away from others while social distancing is home-staying and prohibition of outdoor activities, which has encouraged the use of virtual ways of communication. By returning people to the routine of social life, the importance of practicing physical distancing is being more emphasized. People who are infected by the coronavirus need to self-quarantine for at least 14 days and in this period of time they should stay at home, wash their hands regularly, not share items such as towels and utensils, and not having visitors. In severe cases, hospitalization and intensive care may be required. At the end of the illness, when subjects have no symptoms, with doctor's diagnosis, they can return to normal life. Quarantine has many psychological impacts such as PTSD, anxiety and irritability, insomnia, depression and anger. Also due to the fact that people spend most of their time at home, the risk of intimate partner violence (IPV) in multiple domains of abuse has increased (15–19), however its benefits typically outweigh these health issues when setting public policy. Another important impact of staying at home is increasing the usage of Internet both for telecommuting and browsing for information on outbreaks and other news related to the disease such as the mortality rate (20); which can also be a trigger to the fear of COVID-19 and obtaining incorrect information (9). Besides the concern of the COVID-19 pandemic, Internet, social media and games have become an integral part of individual's lives; which has added a disorder called Internet addiction into the list of problems and psychiatric disorders (21). Addiction is defined as a high dependency on something and the inability to control the consumption that can involve some kinds of substance, behavior and process (22) such as gambling, excessive sexual behavior, compulsive buying, Internet use, or stealing (23). According to the recent statistics, about 4.66 billion people are active internet users as of February 2021, where 3.96 billion people are also active social media users (24). As of April 2019, Iran ranks first in the Middle East with 62.7 million internet users (25) and according to the report of Internet World Stats, it is the 17th country with the greatest number of internet users worldwide.

Over the last decade, increasing population size and the frequency of internet use has become a concern of the possible negative consequences of overuse (26). This concern has increased during the time of the COVID-19 pandemic due to social contact restrictions and the reduction of non-virtual communications and outdoor activities (12). There are some psychological factors which can predict addiction to the internet; such as loneliness, self-esteem and life satisfaction (27), shyness and locus of control (28), depression (29), emotional regulation (30), and self-compassion (31).

The concept of self-compassion was created in response to criticisms of the concept of self-esteem as a component of psychological health. As self-esteem is based on the performance of others, kind of social judgment and comparison, self-efficacy, true self-esteem, self-respect, and self-compassion have been identified as components that provide a better explanation for mental health. Self-compassion is a concept that consists of three parts: (a) kindness toward oneself rather than self-blaming and being self-judgmental during times of difficulty, (b) having human commonalities instead of a sense of isolation and (c) mindfulness vs. over-identification or avoidance toward painful feelings. Being self-compassionate is being used for one who understands his/her condition in a non-evaluative manner and keeps being empathic instead of over criticizing. The person interprets the situation as an experience which may occur to everyone during their lifetime, acknowledging that suffering and he is not the only person in pain in the world. Furthermore, he can keep thoughts and emotions in balanced awareness instead of attaching to one and avoiding the others (32). The relationship between self-compassion and anxiety, depression and self-criticism are negatively significant, while the positive association between self-compassion and wellbeing, optimism and happiness are proven. There is a negative relationship between internet addiction and depression and lower self-esteem thus self-compassion can play a protective role against this psychopathology (33).

Another factor that can predict internet addiction is cognitive emotion regulation which is a general term that is defined as the human's ability to manage and modulate emotions in every difficult situation of life, consciously or unconsciously (34). According to Gross' model, emotion regulation includes 5 stages: (1) situation selection, (2) situation modification, (3) attention deployment, (4) cognitive change and (5) response modulation (34). Moreover, various studies have introduced different emotion regulation strategies that fall into two categories: adaptive and maladaptive. Maladaptive strategies include repression, avoidance, and mental rumination; which are associated with a variety of disorders such as anxiety and depression. Adaptive strategies include problem solving (ability to change conditions that create undesirable emotions), acceptance (accepting emotions and feelings as they are) and reappraisal (positive interpretation of stressful situations as a way of anxiety reduction) (34, 35). Inability to use healthy strategies to moderate negative emotions may lead to many mental disorders such as affective and anxiety disorders; while adaptive ways of emotion regulation are linked to psychological and physical wellbeing (35). Additionally, some research shows that students with severe internet addiction have greater difficulties in emotion regulation (36) and it may be an important variable in understanding the relationship between mental health problems and improper use of social media (37). Other research suggests that activation of maladaptive coping strategies such as rumination, may increase the likelihood of using the Internet as a means of cognitive-emotional self-regulation. Thus, using the Internet may become a strategy for controlling unwanted negative emotions (38).

In general, the pandemic of COVID-19 has affected every part of our lives, on top our psychological health which can be influenced by some non-mental components and some interpersonal issues. Besides that, people are constantly worried about getting infected, whether themselves or their loved ones, so this fear and anxiety has become an integral part of their lives. People may cope with this pressure in different ways; some by exercising at home, some through learning new skills, and some people may spend most of their time in cyberspace, computer games, and more generally, on the Internet. In order to help with the current situation, this work intends to investigate the relationship between anxiety and fear caused by the COVID-19 disease, and Internet addiction with the mediating role of self-compassion and cognitive emotion regulation.

## Methods

### Samples

The target sample in this research was students from different academic levels which were selected using the convenience sampling method. They were invited to participate in this research through popular social media pages and groups. Due to the prevalence of the coronavirus and the need to follow health protocols, online methods were used to collect data in this study. Questionnaires were sent to the target population, through programs such as WhatsApp, Telegram and Instagram. The survey was started in January 2020 and the data collection was done after 2 months. Inclusive criteria are students and those who have access to the internet in order to fill out a questionnaire online. If a questionnaire was not completely done, or only one option had been selected in all questions, the person was excluded from the sample. The questuionnaire was sent to more than 1,200 students and 1,008 of them filled the inclucive criterias. Participation or non-participation in the study was not beneficial or harmful for individuals and all of them answered the questionnaires based on personal satisfaction.

In this study, 12 samples for each subscale were collected. This number of samples required is based on the book of multivariate regression in behavioral research written by Kerlinger (39), which indicates the need for 12 or 15 samples per subscale in this method of analysis. With a total of 18 subscales, there was a requirement to collect data from at least 216 students.

## Materials

### Corona Disease Anxiety Scale

The CDAS has recently been developed and validated to measure anxiety caused by the outbreak of coronavirus in Iran. The final version of this questionnaire has 18 items and 2 components. Items 1 to 9 measure psychological symptoms and items 10 to 18 measure physical symptoms. This tool is scored in a 4-point Likert scale (never = 0, sometimes = 1, most times = 2 and always = 3). High scores in this questionnaire indicate higher levels of anxiety in the individuals. The reliability of this tool was obtained using Cronbach's alpha method for the psychological symptom α = 0.879, the physical symptom α = 0.861, and the whole questionnaire α = 0.919 (40).

### Young Internet Addiction Test (IAT)

The IAT is a 20-item questionnaire that assesses the person's performance at work, school and home (3 questions), social behaviors (3 questions), emotional communication and response via the Internet (7 questions), and general patterns of Internet use (7 questions) (41). Respondents answer on a 5-point Likert measure (“does not apply” to “always”), which people score from 0 to 100. Those who get <49 will be in the “average users”' category, participants scoring between 50 and 79 are “problematic internet users,” and those scoring 80 and above be categorized as “severely problematic users.” In the study of Widyanto et al. (42), the internal validity of the questionnaire was higher than 0.92 and the validity of the retest was also reported to be significant. It also shows good to moderate internal consistency and, alpha coefficients of 0.82 (42). In a Persian psychometric survey of the test, the validity of the retest was 0.82 and internal consistency, where the alpha coefficient was 0.88 (43).

### Self-Compassion Scale Short-Form

The Self-Compassion Scale (SCS) is a 26-item questionnaire with six subscales consist of self-kindness, self-judgment, common humanity, isolation, mindfulness and over-identification; which is a valid and reliable test (44). The Self-Compassion Scale Short-Form (SCSSF) is a shorter 12-item questionnaire and with a 5-point Likert measure that is a reliable and valid alternative to the full version with a high correlation (*r* ≥ 0.97). The internal consistencies for the SCS–SF subscales were 0.54 and 0.75 for the English version of SCS–SF. Reliabilities for all but one subscale (self-kindness) were above 0.60, and Cronbach's alphas of 0.60 and above are acceptable (45). In the Persian version of the test, Cronbach's alphas of 0.91 for the whole scale and 0.77 to 0.92 for the six subscales were calculated. Validity coefficient with the general health questionnaire was−0.45 and for the subscales from−0.28 to−0.48 (46).

### Cognitive Emotion Regulation Questionnaire

The CERQ is a 36-item multidimensional questionnaire designed to identify cognitive emotion regulation strategies that people use in stressful, threatening or traumatic life events; which is a valuable and reliable tool. This questionnaire examines 9 cognitive strategies for emotion regulation (self-blame, blaming others, acceptance, refocusing on planning, positive refocusing, rumination, positive reappraisal, putting into perspective, and catastrophizing) (47). Moreover, the short-form of cognitive emotional regulation (CERQ-short) is an 18-item questionnaire with high alpha reliabilities. Self-blame has the lowest alpha in this questionnaire between the subscale (0.67) and the rest of the alphas were in a range of 0.73 to 0.81 (48). Based on the standardization done in Iran, this questionnaire with Cronbach's alpha between 0.68 and 0.82 (for 9 subscales) has a good validity in the Iranian society (49).

## Result

The research is a cross-sectional and modeling method using SPSS Statistics v22 and AMOS v22 has been applied to analyze the data. Also a description of the demographic information of the participants is given in Table 1.

**Table 1:** Demographic characteristics of study sample (*n* = 1,008).

**Variable**	**Frequency**	**Percentage (%)**
**Gender**		
Male	284	28
Female	724	72
**Education**		
Diploma	360	35
Bachelor	340	34
Masters	236	24
Doctorate	72	7
**Marital status**		
Single	857	85
Married	137	13.5
Divorced	13	1.5
Widowed	1	0.1
**Employment status**		
Physical presence at work	185	18
Teleworking	217	22
Unemployed	606	60
**Type of employment**		
Unemployed	630	62.5
Part-time	249	25
Full-time	129	12.5
**Income**		
Low	75	7.5
Middle	755	75
Good	178	17.5
**Have you been infected by COVID-19?**		
Yes	189	19
No	819	81
**Has any of your family members or friends been infected by COVID-19?**		
Yes	556	55
No	452	45
**Social distance**		
<2 months	120	12
Between 2 and 5 months	187	18.5
More than 5 months	701	69.5

Descriptive indicators such as mean, standard deviation, range of values, and correlation matrix of the studied variables are reported in Table 2. As can be seen, anxiety and fear of COVID-19 has a positive and significant relationship with both Internet addiction (*r* = 0.32) and maladaptive cognitive emotion regulation strategies (*r* = 0.17) and it has a negative relationship with self-compassion (*r* = -0.25).

**Table 2:** Descriptive statistics and the correlation matrix (*n* = 1,008).

**Variable**	**Mean**	**SD**	**Range**	**1**	**2**	**3**	**4**	**5**
1. COVID-19 anxiety	14.09	9.11	1–54	-				
2. Internet addiction	44.20	14.90	20–100	0.32^**^	-			
3. Self-compassion	36.98	7.82	14–57	−0.25^**^	−0.43^**^	-		
4. Maladaptive strategies	25.19	4.72	8–40	0.17^**^	0.11^**^	0.11^**^	-	
5. Adaptive strategies	29.45	5.98	10–49	0.04	0.04	0.23^**^	0.47^**^	

*r, Pearson's correlation coefficient*;

** *p < 0.01*;

^*^p < 0.05.

**Figure 1:**
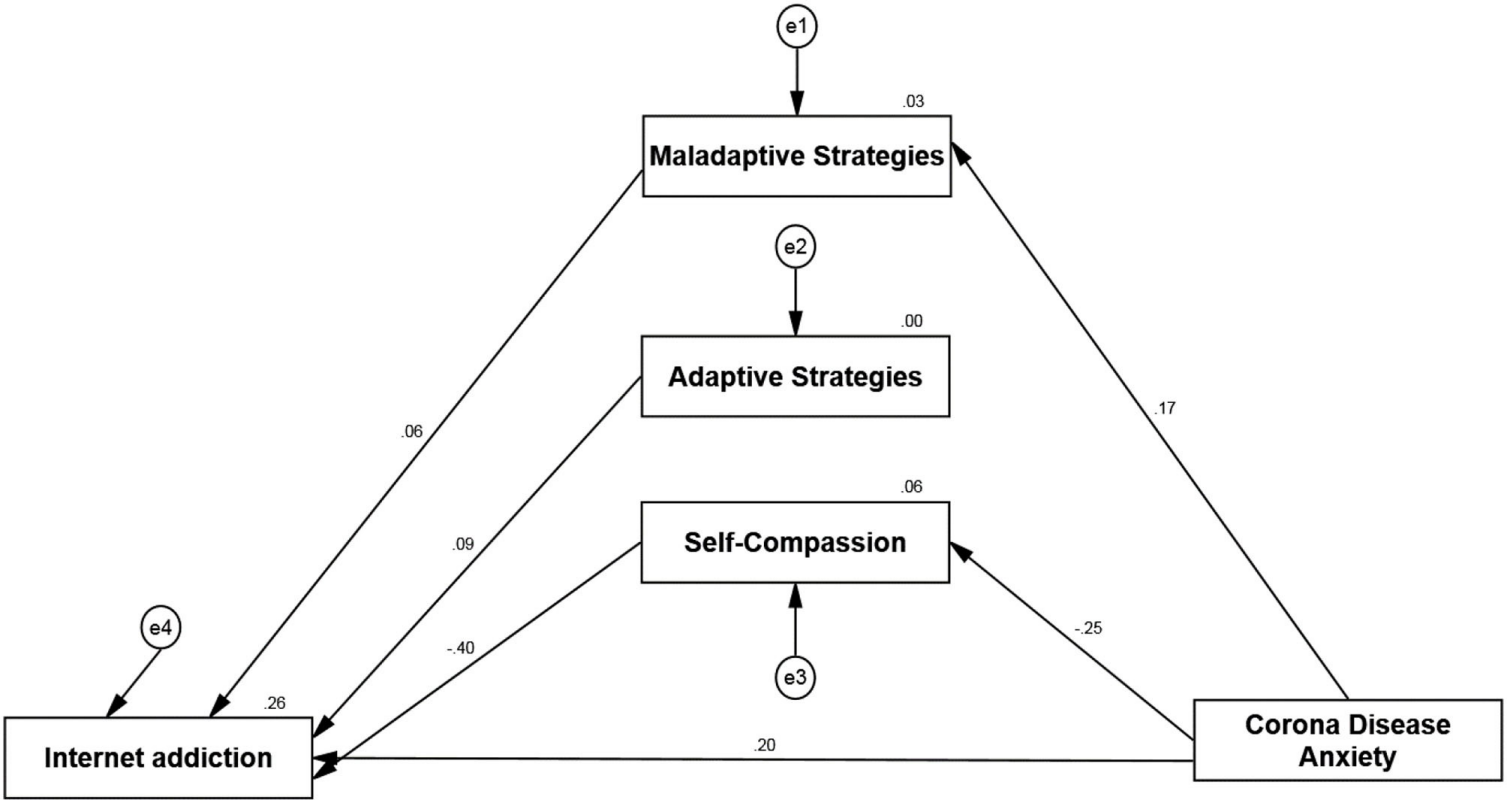
Examining the indirect effect of corona disease anxiety on internet addiction through self-compassion and emotion regulation.

Considering the significant relationships between research variables, the results of path analysis are summarized in Table 3 to investigate the mediating role of self-compassion and cognitive emotion regulation strategies as the role of mediators. The results show that the relationship between all pathways in the mediation model except anxiety and fear of COVID-19 pathway with adaptive cognitive emotion regulation strategies were statistically significant (*p* < 0.0001). Therefore, the findings support the mediating role of self-compassion and maladaptive cognitive emotion regulation strategies in the relationship between anxiety and fear of COVID-19 and Internet addiction. The results are summarized in Figure 1 below. In other words, these findings suggest that people with high anxiety and fear of COVID-19 use maladaptive emotion regulation strategies, which in turn increase their susceptibility to Internet addiction. Also, people with high anxiety and fear of COVID-19 with low levels of self-compassion, are more vulnerable in the path of Internet addiction.

**Table 3:** Summary of mediation analyses on direct and indirect effects of Corona Disease Anxiety on internet addiction (*n* = 1,008).

**Mediator**	**Direct effect**	**Indirect effect via mediator**		
		**Indirect effect**	**Indirect lower CI**	**Indirect upper CI**
Self-compassion	0.36^***^	0.15^***^	0.114	0.206
Maladaptive strategies	0.08^***^	0.03^***^	0.015	0.059
Adaptive strategies	0.03	0.000	0.000	0.000

*CI, 95% confidence interval derived based on 5,000 bootstrapped samples*;

*** *p < 0.0001*.

## Discussion

The aim of this study was to investigate anxiety and fear of COVID-19 as predictors of Internet addiction with the mediating role of self-compassion and cognitive emotion regulation. From the results, it is concluded that in the days when the world is widely affected by COVID-19, there is an association between the fear and anxiety of the virus and the misuse of the Internet. Although the level of anxiety may not indicate that one is suffering from an anxiety disorder, it still requires awareness and, if necessary, intervention. Also, due to the continuing epidemic and its other consequences, people's fear and anxiety may increase in severity to the extent of psychiatric diagnosis. Various factors can be effective in this regard. For example, it seems that limitations related to social distancing, the need to commit to health protocols and high mortality rates, can cause a significant rise in anxiety and fear, which leads to obsessive behaviors such as spending time in cyberspace.

Our findings show that a high level of compassion can be effective in reducing the effect of COVID-19 anxiety on Internet addiction. Since the compassionate person scores higher in the three main indicators of this component, namely self-kindness, human commonalities and mindfulness, it can be inferred as a protective variable, which is congruent with the study of Muris et al. (50, 51). Constantly blaming oneself for the possibility that the individual's actions will put himself or his family members at the risk of infection, as well as feeling responsible for the health of people with whom they are in contact, can cause great anxiety, which is contrary to the constructive effects of self-kindness. Another effect of self-blame is that it leads to the application of maladaptive coping strategies, which is followed by decreased self-esteem, the feeling of helplessness, and social isolation (52). The feeling of common humanity, especially during the coronavirus pandemic, can create this perception that people all around the world are involved in an unavoidable condition, which has imposed many deaths and major limitations in the way of normal life. This factor creates a feeling of closeness to other human beings. Therefore, the less one considers themself a member of human society, the more one will experience anxiety and separation (52, 53). In addition, lack of self-awareness about the present and the constant mental conflict with the issue of coronavirus and fear of death (of themselves and/or their loved ones), and over-identification with these thoughts also increases the level of anxiety. All of these factors explain people turning to virtual networks and the Internet as an inefficient way to deal with this fear and anxiety (12, 54).

Cognitive emotion regulation plays an important role in coping with stressful situations, as it determines the effect of these situations on our mental health. The use of adaptive strategies can help a person cope with stressors such as coronavirus pandemic more efficiently. According to the results, there is a positive relationship between anxiety and fear of coronavirus and the use of maladaptive strategies of cognitive emotion regulation such as avoidance, suppression and rumination, which is consistent with Jungmann and Witthoft (55). Most of people have ruminating thoughts with anxious content such as risk of infection and death of themselves or their loved ones. Moreover, daily exposure to the news of death rates cause people to experience high levels of anxiety. Obsessive use of internet is an avoiding strategy in order to feel less anxious during the pandemic. The negative reinforcing effect of using the Internet turns this behavior into an addiction. Some other reasons for the pathological use of internet could be some dissociative symptoms which are found in their neural pathways (56). It is also proven that social media users have much more social and emotional impairments in comparison with the non-users (57). All these descriptions explain the positive and significant relationship between anxiety and fear of COVID-19 and Internet addiction.

## Conclusions

Due to the increase in addictive behaviors during COVID-19 pandemic (58), self-compassion can play a protective role while maladaptive strategies for emotion regulation such as self-blame, blaming others, and rumination can be risk factors for anxiety and fear of the virus which leads to more obsessive use of internet.

### Suggestions

Self-compassion can be enhanced with treatments such as Mindful Self Compassion (MSC), Compassion Focused Therapy (CFT), Mindfulness-Based Stress Reduction (MBSR), Acceptance and Commitment Therapy (ACT), Dialectical Behavior Therapy (DBT) and Mindfulness Based Cognitive Therapy (MBCT) (59). Also, training emotional regulation skills in limited sessions can help control the level of experienced anxiety. It can also improve adaptive strategies and reduce the use of maladaptive strategies at the time of stress (60). In addition internet is not only the cause of addiction but also due to the extreme relation between anxiety and stress and the use of it, Internet-based interventions could be used to promote wellbeing and manage psychological distress during Covid-19 pandemic (61).

## Limitations

This study was performed on a student population, and precautions should be taken in generalizing the results to other individuals. Also, due to the prevalence of coronavirus, data collection has been done online and by the convenience sampling method, which may bias the results.

## Data Availability Statement

The raw data supporting the conclusions of this article will be made available by the authors, without undue reservation.

## Ethics Statement

Ethical review and approval was not required for the study on human participants in accordance with the local legislation and institutional requirements. Written informed consent for participation was not required for this study in accordance with the national legislation and the institutional requirements.

## Author Contributions

All the data collecting, analyzing, and writing the whole research has been done with the efforts of RM and KP, under the supervision of FL. All authors have read and approved the final manuscript.

## Funding

This research has been written with the efforts of the authors of the article.

## Conflict of Interest

The authors declare that the research was conducted in the absence of any commercial or financial relationships that could be construed as a potential conflict of interest.

## Publisher's Note

All claims expressed in this article are solely those of the authors and do not necessarily represent those of their affiliated organizations, or those of the publisher, the editors and the reviewers. Any product that may be evaluated in this article, or claim that may be made by its manufacturer, is not guaranteed or endorsed by the publisher.
